# Supplementary material for: OTUB1 non-catalytically stabilizes the E2 ubiquitin-conjugating enzyme UBE2E1 by preventing its autoubiquitination
Source: J Biol Chem. 2018 Oct 2;293(47):18285–95. doi: 10.1074/jbc.RA118.004677 (PMC6254341; doi:10.1074/jbc.RA118.004677)
Supplement: Supporting Information [file supp_293_47_18285__index.html]

OTUB1 non-catalytically stabilizes the E2 ubiquitin-conjugating enzyme UBE2E1 by preventing its autoubiquitination — OTUB1 non-catalytically regulates the stability of UBE2E1 — OTUB1 non-catalytically stabilizes the E2 ubiquitin-conjugating enzyme UBE2E1 by preventing its autoubiquitination — OTUB1 non-catalytically regulates the stability of UBE2E1 — Supporting Information 

# OTUB1 non-catalytically stabilizes the E2 ubiquitin-conjugating enzyme UBE2E1 by preventing its autoubiquitination

## Supporting Information

- Raw mass spectrometry data from OTUB1-/- MEFs. - Ratios of proteins in wild-type versus OTUB1-/- MEF cell lysates determined by TMT mass spectrometry analysis. Spreadsheet contains peptides and coverage for each protein identified. Ratios are shown for all three biological replicates.
- Supplementary Information - Supplementary Table 1 Supplementary Figures S1 - S5
