# Supplementary material for: OTUB1 non-catalytically stabilizes the E2 ubiquitin-conjugating enzyme UBE2E1 by preventing its autoubiquitination
Source: J Biol Chem. 2018 Oct 2;293(47):18285–95. doi: 10.1074/jbc.RA118.004677 (PMC6254341; doi:10.1074/jbc.RA118.004677)
Supplement: Supporting Information [file supp_RA118.004677_139149_1_supp_212450_pfz7x8.pdf]

Supplementary Information – Pasupala et al.

(Table 1 – see separate .xls file)

Table 2

Figures S1 – S5

Supplementary Table 2

List of primers for OFF target screening

	FWD primer	REV primer
1	CCACTAGGGGTGGGGCCACA	TCAGTGGAGGAAGAAGCCTG
2	CCAAGGTGCAGGAGGTATGA	ATTGCTTGAAACCGGGCGGC
3	GACTCCGCTATGACAGCACC	TCAGGCCTCCTTCCTGCTGG
4	GACCAGTGGAGGAAGGATTTGAGGAG	GTGCTCAGGGTCCTCCAGGGGTACC
5	GTGCCCCGAGAGCCAAGAAGGC	GAAGCGACGGTTGCCGGGAAGC
6	CCTCCAGCAACTCCCTCACCAC	CGAAGACTAACAGGCCCACATG
7	GGTCATATAATACATTGTGGAGA	CTTAAATGTGGCCAGCACCAGAC
8	GATTGGGGCTGATTTGGGAAGGA	CAAGGCTGGTTCATGTGGCCTAC
9	GCATCTTCACAGACTGTTGTAGTTC	GATGAGGAAACTGGGACCCAAGGCGC
10	GCTGTTACTGTCAGCAGAGGAGCCAG	GGCACAGGGAAGAAGGCAGGCAGATC
11	GCCATAGGAACACTTCTGTCTAC	GTCATTTCACTTAACACATTCAAAT
12	GCCATGGGCTTCCCTTTGTCCCCT	GGAGAGGGTGGCTGGGTGGCCAGC
13	GGTTTCGGCGTGCCAACAGCGAG	GCCAGCGGTGGGAAATGCTTGCC
14	GGCCGGGTGGAGGGCGGGGGTCCG	CCAGGGCCAGAACCCAGCACTATAGC
15	GGAAGCCCCACCCCTGCCTGGGAGG	GGAGCTGGCGTAGGCCCTCCTGACC
16	GAGACCCTATGAATGTAATGAATG	GAGGTTTCAATGTTACATTCCCAC
17	GAGTGAGACTGGTGAGCTGCCTTC	CAGGGGAGGCCGTGGTGACCGGTCC
18	GCAGCAGTATTTGTTTGCTTTGGGC	CATTCTCTAGCATTAGAAGTGGAGC
19	GTTGCTGCCCTGATTCTTGCATTGGA	TCCAGGCCCTACTGTAGGCGGCAGTG
20	CGACGGTTGCCGGGAAGCGCGCGCC	CGAGAGCCGGGAGGGCGGGAGCGAGC
21	GTGGCTATCCCGGCAGGCTCTACCTT	GTTATTCCCCAGCTTCGCCTCACGC
22	CAGTTTTCCATCTGTACTTGTTATG	GCCTGTGCAGACATCTGCCCAGTTG
23	GGTCCACTTCCTGGAGTACACAGA	GTCTCGAACTCCTGACCTTGTTATC
24	GTGTCTCTTTGAGGGTGTGTGTGCC	GGAGAGTGAGGCCCTGGAATGGGCC

Supplementary Figure S1

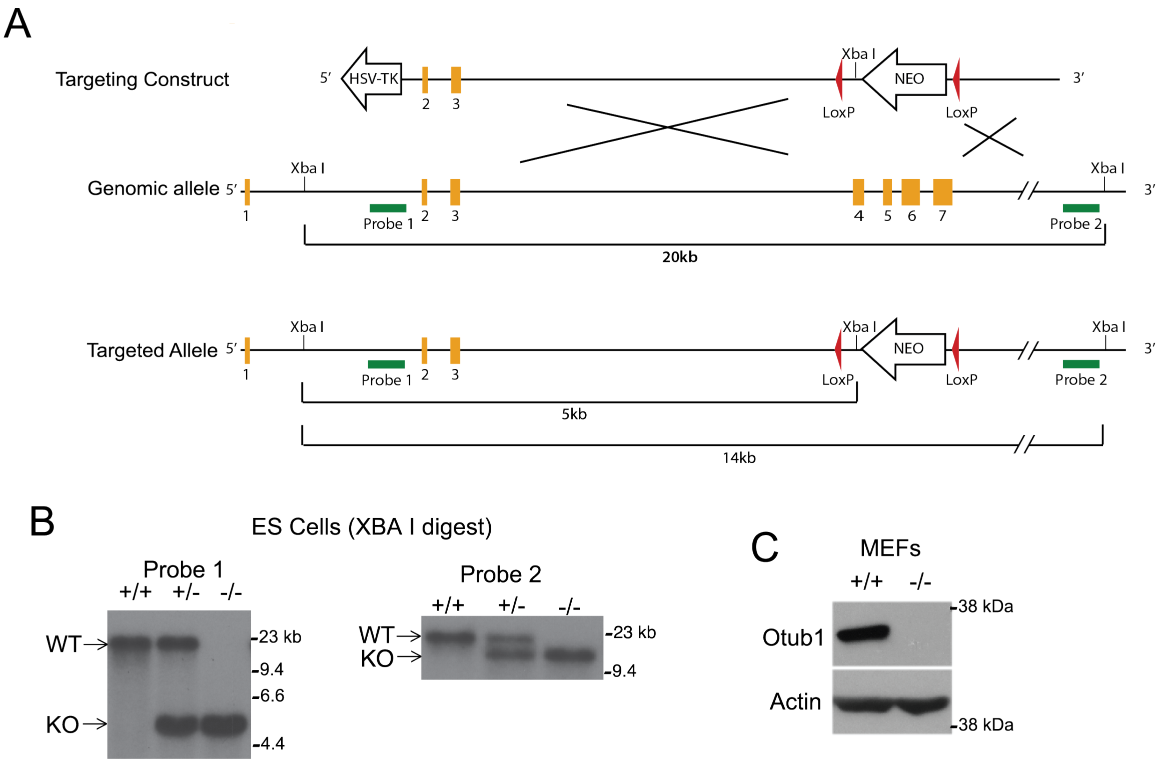

Figure S1: Gene targeting strategy to generate OTUB1 knockout mice. (A) Schematic representation of the gene targeting construct and screening strategy to delete exons 4-7 of OTUB1. (B) Southern blots of Xba I digested genomic DNA from targeted ES cells. (C) Immunoblot of primary MEFs cultured from OTUB1$^{+/+}$ and OTUB1$^{-/-}$.

Supplementary Figure S2

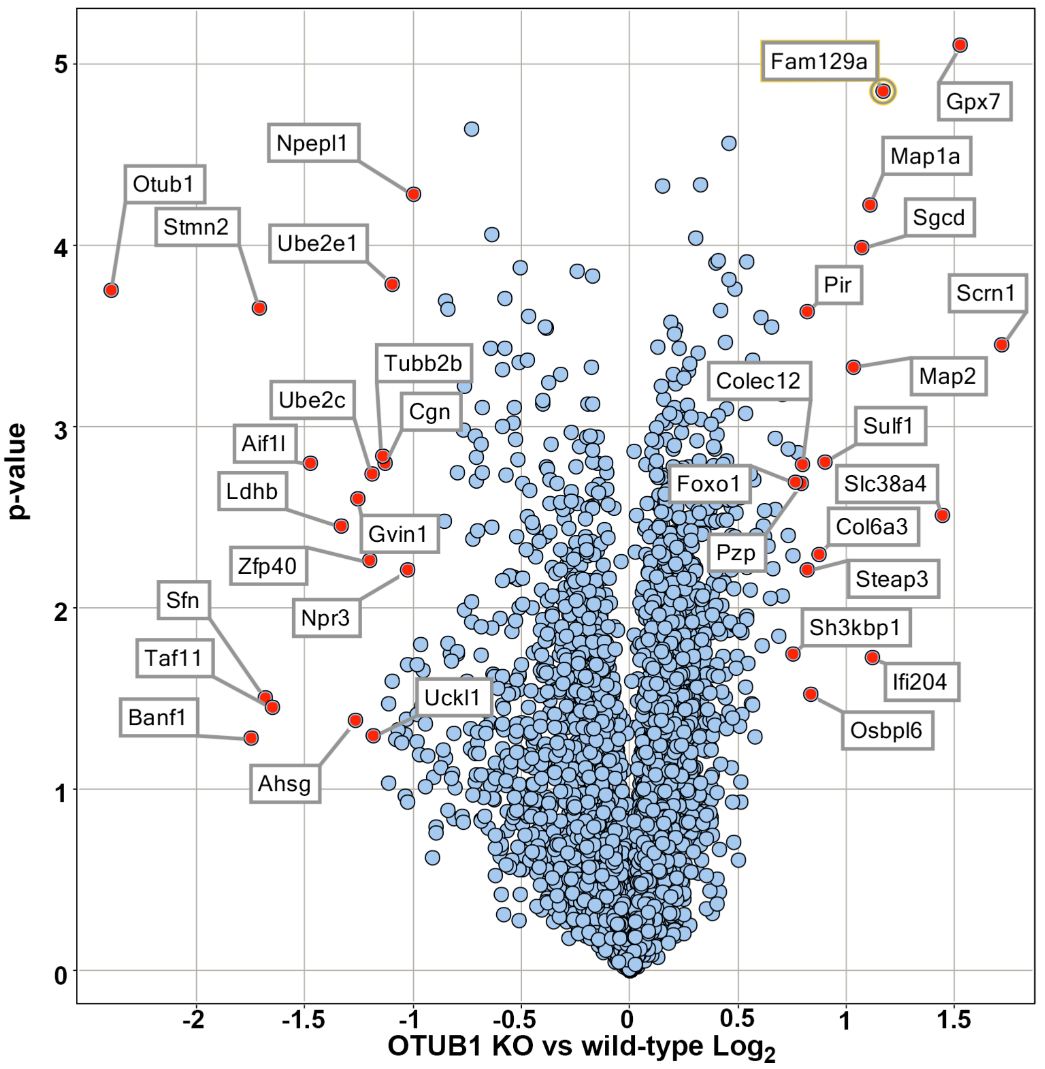

Figure S2: Proteomics analysis of OTUB1$^{-/-}$ knockout MEF cells. Volcano plot of tandem mass tag mass spectrometry analysis of MEF wild type and OTUB1$^{-/-}$ knockout cells showing proteins most affected by the loss of OTUB1.

Supplementary Figure S3

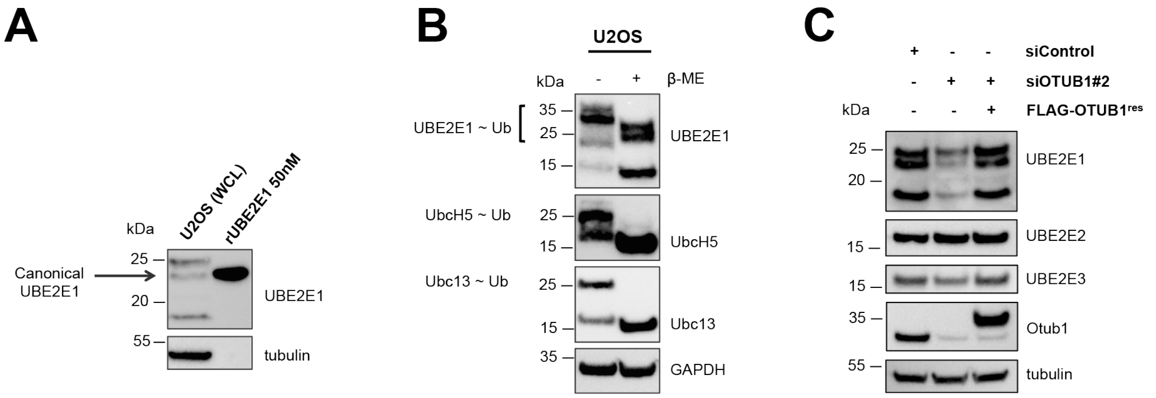

Figure S3: All proteins that cross-react with the anti-UBE2E1 antibody are charged with ubiquitin. (A) Whole cell extract from U2OS cells and 50 nM of recombinant UBE2E1 were analyzed by western hybridization. (B) Whole cell extracts of U2OS cells were incubated with and without β-mercaptoethanol and analyzed by western hybridization with the indicated antibodies. (C) U2OS stable cell lines with control plasmid or FLAG-OTUB1^{res} were transfected with non-target siRNA or individual siRNA against endogenous *OTUB1* gene and assayed for the steady-state levels of indicated proteins by immunoblotting.

Supplementary Figure S4

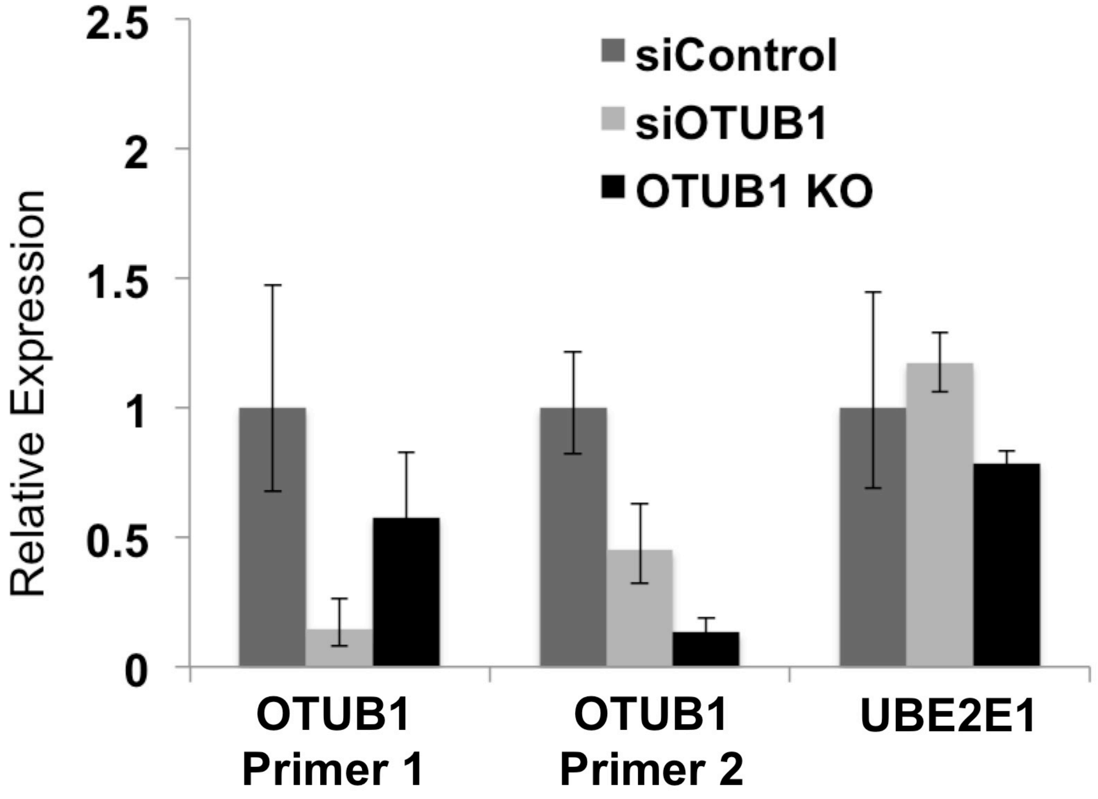

Figure S4: Real-time PCR analysis of OTUB1 and UBE2E1 transcripts in U2OS cells. Total mRNA was extracted from wild-type, siOTUB1, and OTUB1 KO U2OS cells and was quantified by RT-PCR using primers targeting OTUB1 upstream (primer 1), OTUB1 at the CRISPR indel site (primer 2), and UBE2E1. Expression was normalized to TATA-box-binding protein (TBP).

Supplementary Figure S5

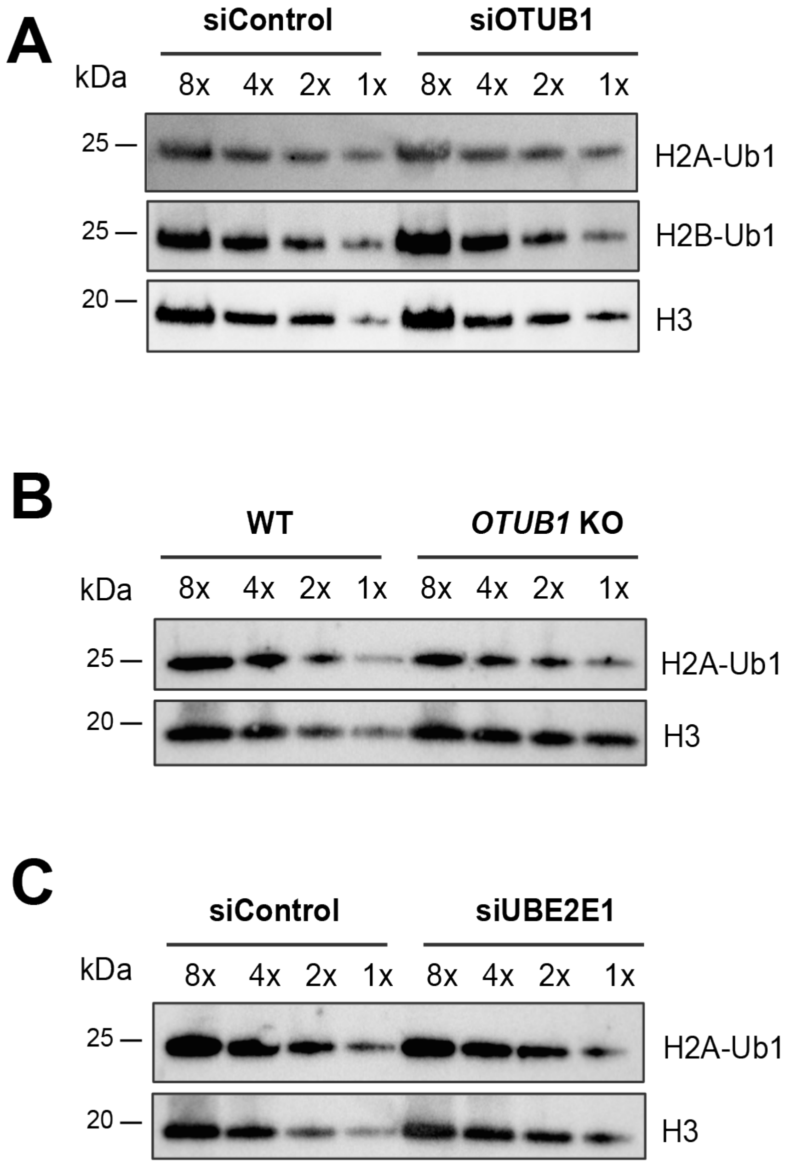

Figure S5: Global levels of monoubiquitinated H2A(K119) in the dynamic range of acid extracted histone from OTUB1 knockdown/knockout and UBE2E1 knockdown U2OS cells. Samples from Figure 6A, 6B and 6C were loaded in gradual two fold dilutions and analyzed by western blotting.
